# A proteomic approach to identify endosomal cargoes controlling cancer invasiveness

**DOI:** 10.1242/jcs.190835

**Published:** 2017-02-15

**Authors:** Jesica Diaz-Vera, Sarah Palmer, Juan Ramon Hernandez-Fernaud, Emmanuel Dornier, Louise E. Mitchell, Iain Macpherson, Joanne Edwards, Sara Zanivan, Jim C. Norman

**Affiliations:** 1 Beatson Institute for Cancer Research, Garscube Estate, Glasgow G61 1BD, UK; 2 Wolfson Wohl Cancer Research Centre, Institute of Cancer Sciences, University of Glasgow, Glasgow G61 1QH, UK; 3 Institute of Cancer Sciences, University of Glasgow, Glasgow G61 1QH, UK

**Keywords:** SILAC, Rab17, Vamp8, Neuropilin-2, Ductal carcinoma *in situ*, Invasive ductal carcinoma, Late endosome, Cell migration, Invasion, Breast cancer

## Abstract

We have previously shown that Rab17, a small GTPase associated with epithelial polarity, is specifically suppressed by ERK2 (also known as MAPK1) signalling to promote an invasive phenotype. However, the mechanisms through which Rab17 loss permits invasiveness, and the endosomal cargoes that are responsible for mediating this, are unknown. Using quantitative mass spectrometry-based proteomics, we have found that knockdown of Rab17 leads to a highly selective reduction in the cellular levels of a v-SNARE (Vamp8). Moreover, proteomics and immunofluorescence indicate that Vamp8 is associated with Rab17 at late endosomes. Reduced levels of Vamp8 promote transition between ductal carcinoma *in situ* (DCIS) and a more invasive phenotype. We developed an unbiased proteomic approach to elucidate the complement of receptors that redistributes between endosomes and the plasma membrane, and have pin-pointed neuropilin-2 (NRP2) as a key pro-invasive cargo of Rab17- and Vamp8-regulated trafficking. Indeed, reduced Rab17 or Vamp8 levels lead to increased mobilisation of NRP2-containing late endosomes and upregulated cell surface expression of NRP2. Finally, we show that NRP2 is required for the basement membrane disruption that accompanies the transition between DCIS and a more invasive phenotype.

## Abstract (precis)

**Highlighted Article:** This paper describes a novel proteomic approach to identify pro-invasive cargoes, which we have used to determine that Rab17 controls Vamp8 levels to drive neuropilin-2 trafficking, promoting invasion.

## INTRODUCTION

The membrane trafficking events controlled by Rab GTPases influence cellular processes that accompany cancer initiation and progression, including loss of cell polarity, and the drive to invasion and metastasis (De Franceschi et al., 2015; Goldenring, 2013). The contribution made by Rab GTPases to acquisition of invasive behaviour has been intensively studied, and the molecular machinery that is responsible for trafficking receptors that control cell adhesion and cell migration is becoming well understood (Miller et al., 2000; Rainero and Norman, 2013). There is good evidence that Rab11 GTPase control of integrin and receptor tyrosine kinase (RTK) recycling drives invasive migration in cancer, and expression of Rab11 isoforms and their effectors, such as Rab-coupling protein (RCP), is linked to metastasis (Caswell et al., 2008). Furthermore, Rab21 is closely associated with integrin trafficking, and the contribution made by this Rab GTPase to cancer invasiveness and suppression of apoptosis is now well documented (Alanko et al., 2015; Högnäs et al., 2012).

Loss of epithelial cell polarity is a key event in cancer progression, and one that is generally accepted to precede acquisition of invasive and metastatic capabilities (McCaffrey and Macara, 2011). For example, in breast cancer, epithelial polarity is progressively lost as tumours progress from pre-malignant lesions to invasive carcinoma. Ductal carcinoma *in situ* (DCIS), which is a heterogeneous group of lesions characterised by intraductal proliferation of malignant epithelial cells, possess an intact basement membrane (which may be considered to be a remnant basolateral domain) but the cells display no functional apical surface (Wiechmann and Kuerer, 2008). The progression of DCIS to invasive ductal carcinoma (IDC) occurs when the basement membrane becomes disrupted and this last vestige of epithelial polarity is lost. Despite the fact that transition between DCIS and IDC is an accepted watershed in disease aggressiveness, the key molecular and cellular drivers of this process are not well understood (Clark et al., 2011; Hannemann et al., 2006; Vincent-Salomon et al., 2008). Since the DCIS to IDC transition involves loss of aspects of epithelial polarity, this is likely to be driven by alterations to Rab GTPase levels and/or function. It is already known that Rab25, a Rab11 GTPase whose expression is largely restricted to epithelia, is often lost in breast cancer (Cheng et al., 2010), and deletion of Rab25 accelerates tumorigenesis in a mouse model of colon cancer (Nam et al., 2010). One interpretation of this is that loss of Rab25 leads to disruption of trafficking pathways that maintain aspects of normal epithelial polarity. Rab17 was the first epithelial-cell-specific small GTPase to be identified, and its expression is induced as epithelial cells polarise (Hunziker and Peters, 1998; Lütcke et al., 1993; Zacchi et al., 1998). There is some debate as to the trafficking pathways controlled by Rab17, but a consensus is emerging that this Rab GTPase is associated with transcytosis of receptors, such as the polymeric immune globulin receptor (dlgA) and the transferrin receptor (TfnR), from the basolateral to the apical surface of epithelial cells (Hansen et al., 1999; Zacchi et al., 1998). We have recently identified Rab17 as a gene that is suppressed during the acquisition of invasive migration, which accompanies upregulation of ERK2 kinase signalling (von Thun et al., 2012). Furthermore, we have shown that Rab17 levels must be suppressed for ERK2 to drive breast cancer invasiveness. Consistently, reduced levels of Rab17 have been shown to be associated with increased aggressiveness in hepatocellular carcinoma (Wang et al., 2015). Taken together, these studies suggest that Rab17 may oppose events that accompany the DCIS to IDC transition – such as the loss of cell polarity and the acquisition of invasiveness – and this is likely to be mediated by controlling endosomal trafficking of pro- and/or anti-invasive receptor cargoes.

As there is no reason to propose that the known receptor cargoes of Rab17-regulated transport – such as dlgA and TfnR – would be drivers of the DCIS to IDC transition or cancer invasiveness, we developed an unbiased mass spectrometry (MS)-based proteomic approach to investigate the Rab17 interactome and the protein cargoes whose trafficking it influences. To do this, we combined quantitative stable isotope labelling with amino acids in cell culture (SILAC)-based proteomics with a biotinylation-based method to separate plasma membrane from endosomal proteins. By using this approach, we found that Rab17 mediates its anti-invasive effects via interaction with the SNARE protein Vamp8, and that Rab17 and Vamp8 influence the endosome–plasma membrane distribution of a number of cargoes that might be expected to influence invasiveness. Prominent amongst these proteins was neuropilin-2 (NRP2), which moves from endosomes to the plasma membrane following Rab17 depletion. We conclude by showing that NRP2 is required for the DCIS to IDC transition and the acquisition of invasiveness that is driven by depletion of Rab17 or Vamp8.

## RESULTS

### Low levels of Rab17 mRNA correlate with poor breast cancer survival

**Fig. 1.**
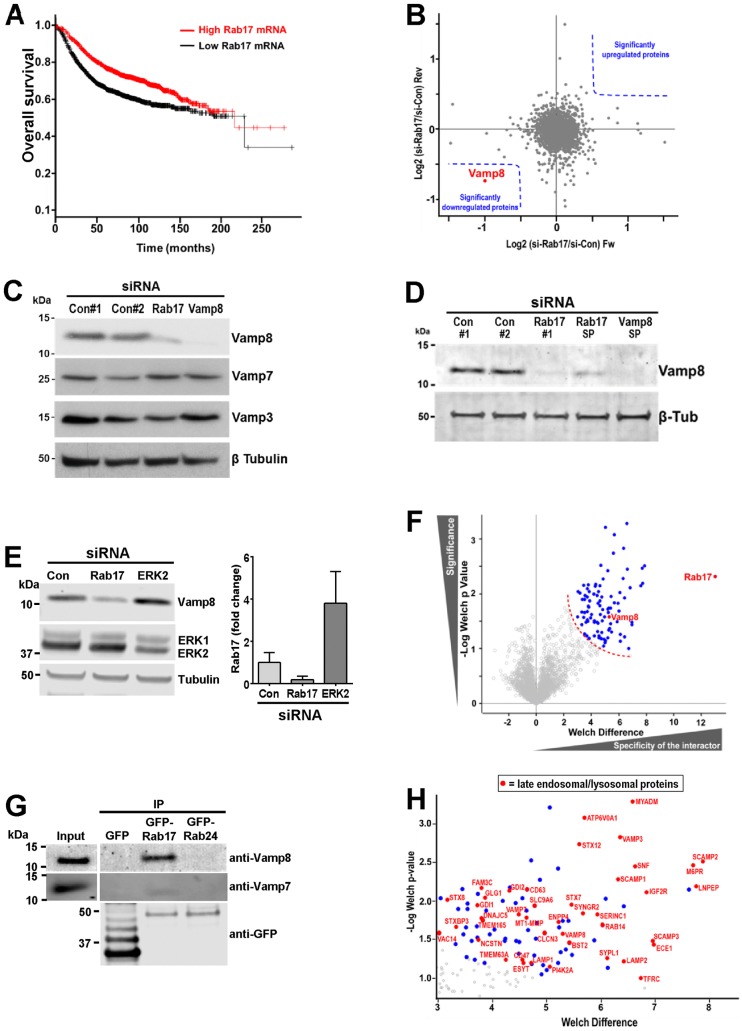
**Rab17 is a key regulator and interacting protein of the endosomal v-SNARE Vamp8.** (A) Kaplan–Meier plot showing the influence of Rab17 expression on overall survival from breast cancer. The red and black lines represent patients with Rab17 expression above and below the median, respectively. *n*=1778 patients (low Rab17); *n*=1776 patients (high Rab17). Log rank test, *P*=1.2×10^−11^. (B) MDA-MB-231 cells that had been SILAC-labelled with heavy- and light-isotope amino acids were transfected with non-targeting siRNA (si-Con) or an siRNA targeting Rab17 (si-Rab17), respectively [forward (Fw) experiment]. For the reverse (Rev) experiment, these labelling conditions were swapped. Scatter plot indicates the SILAC ratio between siRab17 and siCon cells (si-Rab17/si-Con; log_2_ scale) for each protein obtained for the forward versus the reverse experiments for the whole-cell proteome. Blue dotted lines indicate the regions encompassing significantly affected proteins (significance B statistical test, FDR of 5%, Perseus software). (C–E) MDA-MB-231 cells were transfected with siRNAs targeting Rab17 (SMARTPool or individual oligonucleotide Rab17#1), Vamp8 [SMARTPool (SP)], ERK2 or non-targeting controls (si-Con#1 and si-Con#2). Vamp8, Vamp7, Vamp3 and ERK1 and ERK2 expression levels were then determined by western blotting. In E, Rab17 expression was determined by using quantitative PCR (qPCR) relative to that in control (con) (graph). Data are mean±s.e.m. (F) MDA-MB-231 cells were transfected with GFP or GFP–Rab17. GFP-tagged proteins were isolated using GFP-Trap_A agarose beads (Chromotek). Proteins eluted from the beads were resolved by SDS-PAGE, stained with Coomassie Blue (Expedeon) and in-gel digested for MS analysis. Statistical testing of differences between means (Welch’s *t*-test) was performed to show the most differentially expressed proteins across three independent experimental replicates. The red dotted line indicates the significance threshold. Blue dots denote the 95 significant interacting proteins identified. (G) MDA-MB-231 cells were transfected with GFP, GFP–Rab17 or GFP–Rab24. GFP-tagged proteins were isolated as described in A, and immunoprecipitates were analysed by western blotting with antibodies recognising GFP, Vamp8 or Vamp7. IP, immunoprecipitation. (H) Zoom of plot in Fig. 1F showing the identity (gene name) of late-endosome- and lysosome-associated proteins (red dots) in the Rab17 interactome. 43% of the members of the Rab17 interactome belong to the lysosome and late-endosome category, and have been annotated according to Chapel et al. (2013).

We have previously shown that reduced Rab17 mRNA levels are associated with invasive migration of breast cancer cells (von Thun et al., 2012). To determine whether this observation is pertinent to aggressiveness of the human disease, we interrogated the cancer Biomedical Informatics Grid (caBIG), Gene Expression Omnibus (GEO) and The Cancer Genome Atlas (TCGA) repositories using the ‘kmplot’ analysis tool (Győrffy et al., 2013). Consistent with our previous results, we found that low levels of Rab17 expression in breast tumours was strongly associated with poor overall patient survival (*P*<0.0001) (Fig. 1A), and this was statistically significant in all breast cancer intrinsic subtypes (Fig. S1A–C), with the exception of the HER-enriched subtype (Fig. S1D). Survival of ovarian cancer patients was also associated with low Rab17 expression (*P*<0.0001) (Fig. S1E), but we were unable to demonstrate any correlation between Rab17 levels in lung or gastric cancers (data not shown).

### Quantitative proteomics indicate that Rab17 controls Vamp8 levels

We hypothesised that Rab17 may restrict invasiveness by controlling the expression or trafficking of proteins that suppress invasion. To search for these, we knocked down Rab17 (Fig. S1F) and performed a SILAC-based global proteomic MS analysis. We labelled control and Rab17-knockdown MDA-MB-231 breast cancer cells with light-isotope and heavy-isotope SILAC amino acids, respectively (forward experiment), and with heavy-isotope and light-isotope amino acids, respectively (reverse experiment). We then prepared cells extracts and quantified them on an LTQ-Orbitrap. MS data were analysed with the MaxQuant computational platform. Accurate protein quantification highlighted high reproducibility between forward and reverse experiments. Despite the large number of proteins that were unambiguously identified and quantified, we found that only a single protein was significantly downregulated (in both forward and reverse experiments) in Rab17-knockdown cells, and this was the vesicle (v)-SNARE Vamp8 (Fig. 1B; Table S1). We confirmed this result by knocking down Rab17 in MDA-MB-231 cells using either a single small interfering (si)RNA oligonucleotide or pooled siRNAs, followed by western blotting (Fig. 1C,D). Moreover, this analysis indicated that levels of Vamp3 and Vamp7 (the most closely related Vamps to Vamp8) were unaffected by knockdown of Rab17 (Fig. 1C). Furthermore, since Rab17 knockdown suppressed Vamp8 protein but not levels of its mRNA, we conclude that Rab17 controls Vamp8 expression post-transcriptionally (Fig. S2A,B).

Our previous work has identified Rab17 to be a key effector of mitogen-activated protein (MAP) kinase signalling. Following suppression of ERK2 but not of ERK1 (ERK2 and ERK1 are also known as MAPK1 and MAPK3, respectively), Rab17 levels are increased and this leads to increased invasiveness (von Thun et al., 2012). To determine whether the ability of MAP kinase signalling to influence Rab17 results in control of Vamp8 levels, we knocked down ERK2 and measured Vamp8 levels by western blotting. Knockdown of ERK2 led to increased levels of Rab17 mRNA and Vamp8 protein (Fig. 1E), further supporting the view that Rab17 controls Vamp8 levels and suggesting that suppression of this v-SNARE may underlie the ability of ERK2 to drive invasiveness.

### The Rab17–Vamp8 complex is localised to late endosomes

We used MS-based proteomics to investigate the Rab17 interactome. We expressed GFP-tagged Rab17 (GFP–Rab17) or GFP in MDA-MB-231 cells and immunoprecipitated these using GFP-trap beads. A label-free quantification approach was then used to identify a set of 95 proteins that constitute the Rab17 interactome, and Vamp8 was a prominent component of this (Fig. 1F; Table S2). We used western blotting to confirm the association between Rab17 and Vamp8 (but not Vamp7), and also found that Rab24 (a Rab GTPase which is closely related to Rab17) did not coimmunoprecipitate with Vamp8, indicating that there was a degree of specificity to the Rab17–Vamp8 interaction (Fig. 1G). To determine whether Vamp8 and Rab17 are able to interact directly with each other, we produced the cytoplasmic region of Vamp8 (Vamp8^cyto^) and GST-tagged Rab17 constructs (both the wild type and a constitutively active GTPase-deficient mutant of the protein) in *Escherichia coli* and purified these. However, we were unable to demonstrate co-precipitation of purified soluble Vamp8^cyto^ with glutathione-bead-conjugated Rab17 constructs (Fig. S2C), indicating that these proteins either do not interact directly or interact transiently. Next, we used STRING software (http://string-db.org) (Szklarczyk et al., 2015) to examine known functional interactions between proteins of the Rab17 interactome. In addition to the anticipated Rab proteins involved in signal transduction and the SNARE complex groups, we found that Rab17 associated with proteins related to lysosomes (Fig. S2D). Indeed, when we annotated our Rab17 interactome to denote the intracellular compartmentalisation of its components, we found that 43% of the proteins interacting with Rab17 could be classified as late endosomal and/or lysosomal proteins (Fig. 1H).

**Fig. 2.**
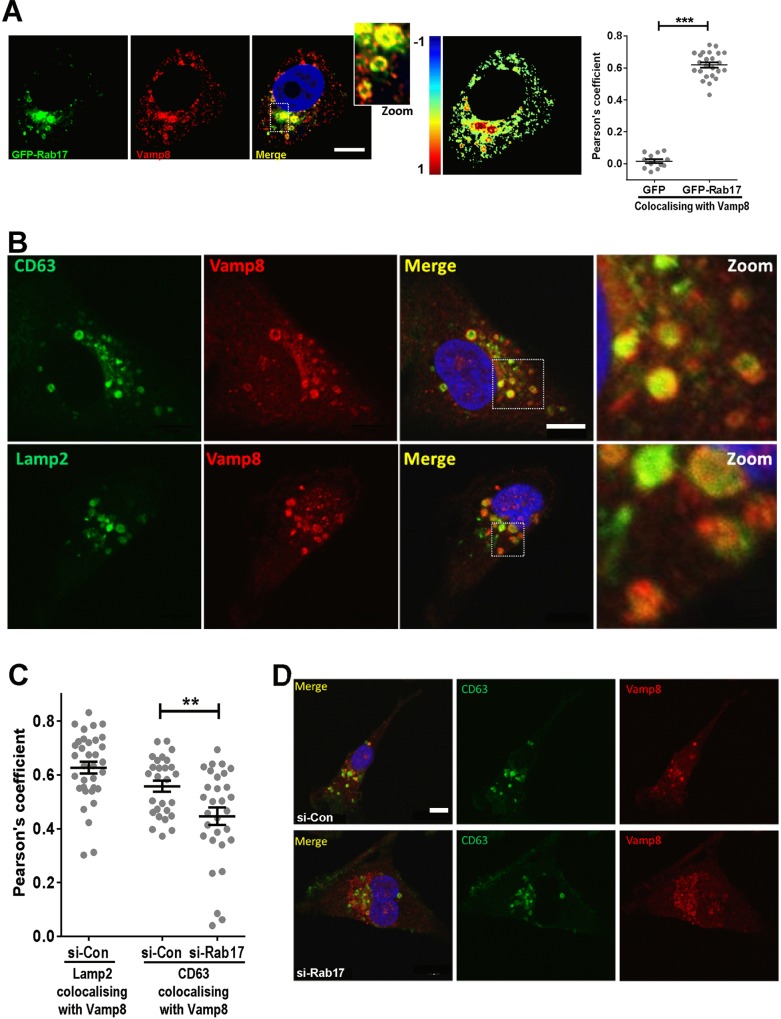
**Rab17 and Vamp8 colocalise at late endosomal membranes.** (A) MDA-MB-231 cells were transfected with GFP or GFP–Rab17 (green signal) and fixed, and endogenous Vamp8 was then visualised using immunofluorescence (red signal). Pearson's coefficients were determined using the ‘Colocalization Colormap’ ImageJ plugin and are depicted both as a pseudocolour image and graphically. Mean±s.e.m. are indicated, number of cells=12 (GFP), 24 (GFP–Rab17). ***P<0.001; U-Mann–Whitney. Scale bar: 10 µm. (B–D) MDA-MB-231 cells were transfected with siRNA targeting Rab17 (si-Rab17) or a non-targeting control (si-Con). Following this, cells were fixed and stained for endogenous Vamp8 (red) and either CD63 (green) or Lamp2 (green). The nuclei were counterstained with DAPI (blue). Dotted squares indicate the zoomed regions. Scale bar: 10 µm. (C) Pearson's coefficients were determined as for A. **P<0.01; U-Mann–Whitney test.

GFP–Rab17 displayed significant colocalisation (Pearson's coefficient=0.62±0.016; mean±s.e.m.) with endogenous Vamp8 at vesicular structures in the perinuclear region of MDA-MB-231 cells, consistent with late endosomal localisation of Rab17 and Vamp8 (Fig. 2A). Indeed, endogenous Vamp8 displayed significant colocalisation with both endogenous (Fig. 2B,C; Pearson's coefficient ∼0.6) and GFP-tagged (Fig. S3) markers of late endosomes, such as CD63, Lamp1 and Lamp2. As described in Fig. 1, we observed a reduction in Vamp8 levels following Rab17 knockdown. Correspondingly, the late endosomal distribution of Vamp8 was disrupted following transfection with siRNA against Rab17. Thus, the residual Vamp8 in Rab17-knockdown cells was distributed more generally in the central region of the cell, and its colocalisation with CD63-positive structures was significantly reduced (Fig. 2C,D).

### Rab17 and Vamp8 oppose breast cancer cell invasiveness in 3D models

**Fig. 3.**
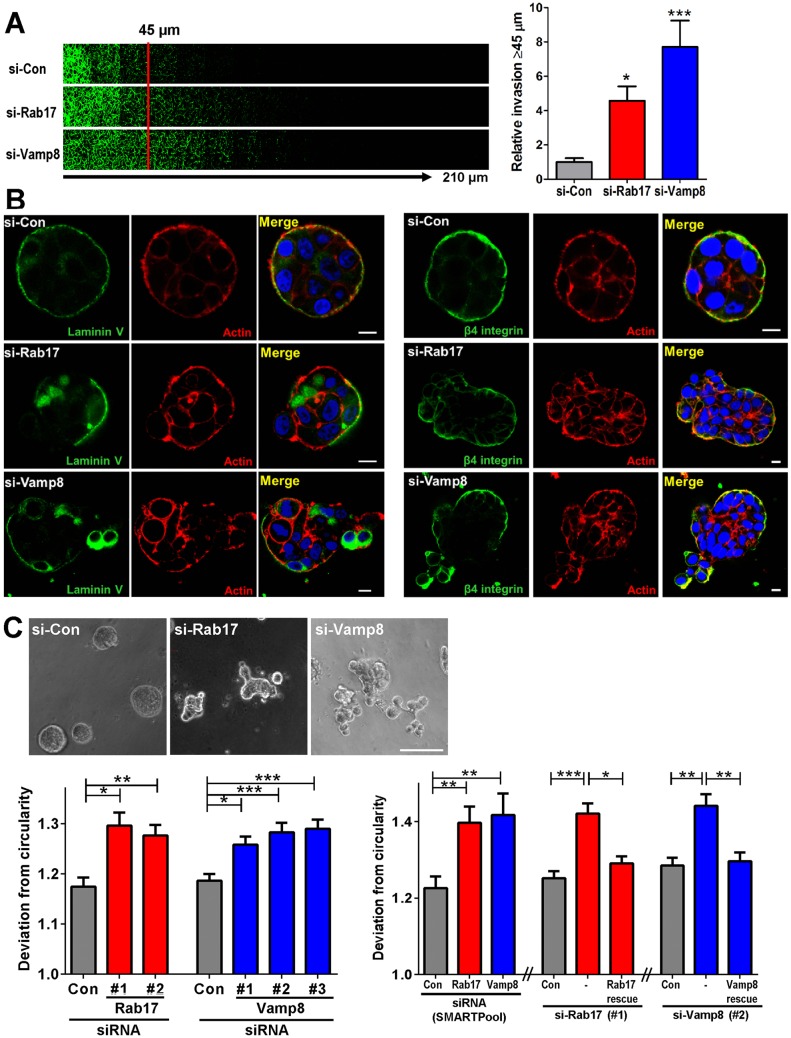
**Rab17 and Vamp8 oppose breast cancer cell invasiveness.** (A) MDA-MB-231 cells were transfected with siRNAs targeting Rab17 (si-Rab17) or Vamp8 (si-Vamp8), or a non-targeting control (si-Con). Invasiveness into fibronectin-supplemented Matrigel was determined using an inverted invasion assay. Invading cells were stained with Calcein-AM and visualised by confocal microscopy. Serial optical sections were captured at 15 µm intervals and are presented as a sequence in which the individual optical sections are placed alongside one another with increasing depth from left to right, as indicated. Migration was quantified by measuring the fluorescence intensity of cells penetrating the Matrigel to depths of 45 µm and greater, and expressing this as a percentage of the total fluorescence intensity of all cells within the plug. Values are mean±s.e.m. *n*=12 measurements from three independent experiments. Kruskal–Wallis (*P*<0.0001) and Dunn's test for the multiple comparisons were performed (*P<0.05; ***P<0.001). (B,C) MCF10DCIS.com cells were transfected with siRNAs targeting Rab17 [si-Rab17 (SMARTPool) or individual siRNA sequences Rab17#1 or Rab17#2] or Vamp8 [si-Vamp8 (SMARTPool) or individual siRNA sequences Vamp8#1, Vamp8#2 or Vamp8#3], or a non-targeting control (si-Con), in combination with plasmids encoding siRNA-resistant forms of GFP–Rab17 (Rab17-rescue) or GFP–Vamp8 (Vamp8-rescue) as indicated. Cells were plated into Matrigel and cultured for 3 days, following which, cells were fixed, and laminin-V and β4 integrin were visualised by immunofluorescence (B; green signal). The actin cytoskeleton (red signal) and nuclei (blue signal) were visualized with phalloidin–Alexa-Fluor-546 and DAPI staining, respectively (B). Scale bars: 10 μm (B). In C, acini were visualised using phase contrast microscopy. Circularity was determined by measuring the ratio between the width and length of each acini. Values are mean±s.e.m., *n* values in C from left to right are: *n*=88 non-targeting control, (Con), *n*=160 (Rab17#1), *n*=130 (Rab17#2), *n*=243 (Con), *n*=293 (Vamp8#1), *n*=222 (Vamp8#2), *n*=220 (Vamp8#3), *n*=168 (Con), *n*=174 (Rab17), *n*=201 (Vamp8), *n*=63 (Con), *n*=51 (Rab17), *n*=39 (Vamp8), *n*=177 (Con), *n*=253 (Rab17#1), *n*=270 (Rab17#1+ Rab17-rescue), *n*=173 (Con), *n*=174 (Vamp8#2) and *n*=176 (Vamp8#2+ Vamp8-rescue). Scale bar: 100 µm. Kruskal–Wallis and Dunn's test for the multiple comparisons were performed (***P<0.001, **P<0.01, *P<0.05).

Our identification of Vamp8 as an interactor of Rab17 and the fact that Vamp8 was downregulated in Rab17-depleted cells prompted us to determine whether Vamp8 contributes to the ability of Rab17 to function as a suppressor of breast cancer cell invasiveness. Initially, we investigated the consequences of knocking down Rab17 or Vamp8 on the ability of MDA-MB-231 cells to invade into plugs of Matrigel that had been supplemented with fibronectin. siRNA-mediated knockdown of Rab17 increased invasiveness of MDA-MB-231 cells. Interestingly, siRNA knockdown of Vamp8 also promoted invasiveness into Matrigel plugs, consistent with the view that Vamp8 is functionally linked to Rab17 (Fig. 3A). These data led us to hypothesise that Rab17- and Vamp8-dependent trafficking might drive the transition between DCIS and IDC in the mammary gland, thus contributing to breast cancer progression. Experimentally, this transition may be modelled using the MCF10DCIS.com breast cancer cell line. MCF10DCIS.com cells are estrogen receptor (ER)-negative premalignant mammary carcinoma cells derived from the ‘normal’ MCF10A cell line and are known to form well-defined comedo-like DCIS structures when injected as subcutaneous or intraductal xenografts (Behbod et al., 2009; Miller et al., 2000). However, with time, these lesions spontaneously progress to invasive carcinoma, characterised by disruption of their surrounding basement membrane and the development of invasive outgrowth. Elements of this progression may be recapitulated in three-dimensional (3D) culture (Jedeszko et al., 2009). Since the levels of Vamp8 in MCF10DCIS.com cells were dependent on Rab17 expression (Fig. S4A,B), we used this model to study the role of Rab17 and Vamp8 in the DCIS to IDC transition. When MCF10DCIS.com cells were cultured for 3 days in Matrigel, they formed well-organised comedo-like structures. These were surrounded by basement membranes as evidenced by immunofluorescence staining for the basolateral marker β4 integrin and the basement membrane component laminin-V (Fig. 3B). We quantitatively assessed the shape of these organoids and found that MCF10DCIS.com cells formed structures that were roughly spherical, as reflected by a low deviation from circularity of a cross-sectional focal plane (Fig. 3C). However, when either Rab17 or Vamp8 were knocked down in MCF10DCIS.com cells (using either a SMARTPool or individual siRNA sequences), structures with a high degree of sphericity were able to initially form (data not shown), but their symmetry became significantly disrupted after 3 days of culture in Matrigel (Fig. 3C). Consistently, immunofluorescence staining for β4 integrin and laminin-V indicated that siRNA knockdown of Rab17 or Vamp8 drove substantial disruption of the basement membrane surrounding the comedo-like structure, allowing MCF10DCIS.com cells to migrate out of the organoid (Fig. 3B). Furthermore, the ability of siRNAs targeting Rab17 or Vamp8 to disrupt the sphericity of MCF10DCIS.com organoids was completely inhibited by expression of siRNA-resistant (rescue) versions of GFP–Rab17 and GFP–Vamp8, respectively (Fig. 3C; Fig. S4C,D). Finally, combined knockdown of Rab17 and Vamp8 did not increase the disruption of sphericity that was evoked by knockdown of these proteins individually, consistent with Rab17 and Vamp8 operating in the same signalling axis (Fig. S4E). Taken together, these data are consistent with a role for Vamp8 in mediating the anti-invasive functions of Rab17, and this is evident in a 3D model of the DCIS to IDC transition.

### Vamp8 levels are associated with tumour grade in breast cancers

**Fig. 4.**
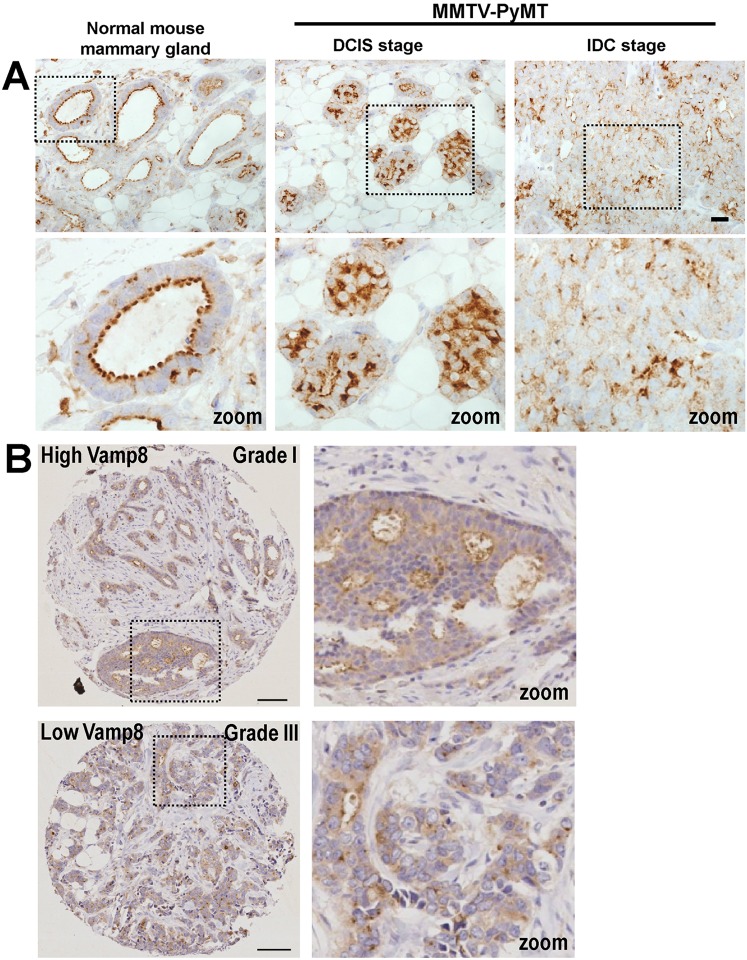
**Low Vamp8 levels are associated with high tumour grade in breast cancer.** Vamp8 (brown staining) was visualised in paraffin-embedded sections of normal mammary ducts and mammary gland tumours from MMTV-PyMT mice (A) and in a human breast cancer TMA (B) using immunohistochemistry. Dotted squares indicate the zoomed-in regions shown in the bottom row of A and the right-hand column of B. Scale bars: 25 µm (A); 100 μm (B). In A, the tumours depicted are from the intraepithelial neoplasm or DCIS (centre panels), and the invasive ductal carcinoma (right panels) stage of the disease. In B, representative examples of a low-grade (Grade I) tumour with intense Vamp8 staining (left panels) and a high-grade (Grade III) tumour with less-intense Vamp8 staining (right panels) are displayed.

Our data, indicating the likelihood that Rab17 exerts its anti-invasive effects by maintaining Vamp8 levels, prompted us to investigate the relationship between Vamp8 and invasive pathology in breast cancer. In the normal mouse mammary gland, Vamp8 appeared to be associated with the luminal face of the ductal epithelium (Fig. 4A). Expression of the polyoma middle-T antigen (PyMT) under control of the MMTV mammary-specific promoter (MMTV-PyMT) results in formation of tumours in the mouse breast with high penetrance. MMTV-PyMT tumours have a gene expression profile similar to that of human luminal-B cancers (Herschkowitz et al., 2007), and most of these progress from DCIS-like structures to IDC in a similar manner to the human disease. We, therefore, looked at the distribution of Vamp8 in a number of tumours from MMTV-PyMT mice and compared this with their histopathology. We found that Vamp8 levels were high in the less-advanced MMTV-PyMT tumours that displayed DCIS-like pathology, whereas in more invasive tumours with IDC-like characteristics, Vamp8 levels were low (Fig. 4A). We then deployed a large tissue microarray (TMA) containing 542 human breast tumours to test whether this inverse relationship between Vamp8 levels and features of tumour aggressiveness could be observed in human breast cancer. Furthermore, because some Vamp8 immunoreactivity observed in breast tumours appeared to be diffusely distributed within the cells, we scored our TMA only for Vamp8 that was localised to membranous structures that were either in the cell periphery or within the cell. There was a highly significant inverse correlation between membranous Vamp8 staining and tumour grade. Indeed, the majority of low-grade (grade I) tumours had high levels of Vamp8, whereas most high-grade (grade III) tumours displayed less-intense membranous Vamp8 staining (Table 1; Fig. 4B). Moreover, Vamp8 levels were significantly correlated with the breast cancer molecular subtype in a way that was consistent with tumour grade. For example, Vamp8 levels were low in the majority of the highly aggressive ER-negative subtype, whereas a minority of ER-positive tumours had high Vamp8 levels (Table 1). These data indicate that loss of Vamp8 occurs as murine mammary tumours progress from DCIS to IDC and that Vamp8 levels are maintained in less-aggressive luminal-A and low-grade human tumours in comparison to more-aggressive cancers, which is consistent with an anti-invasive role for Rab17 and Vamp8.

**Table 1.**
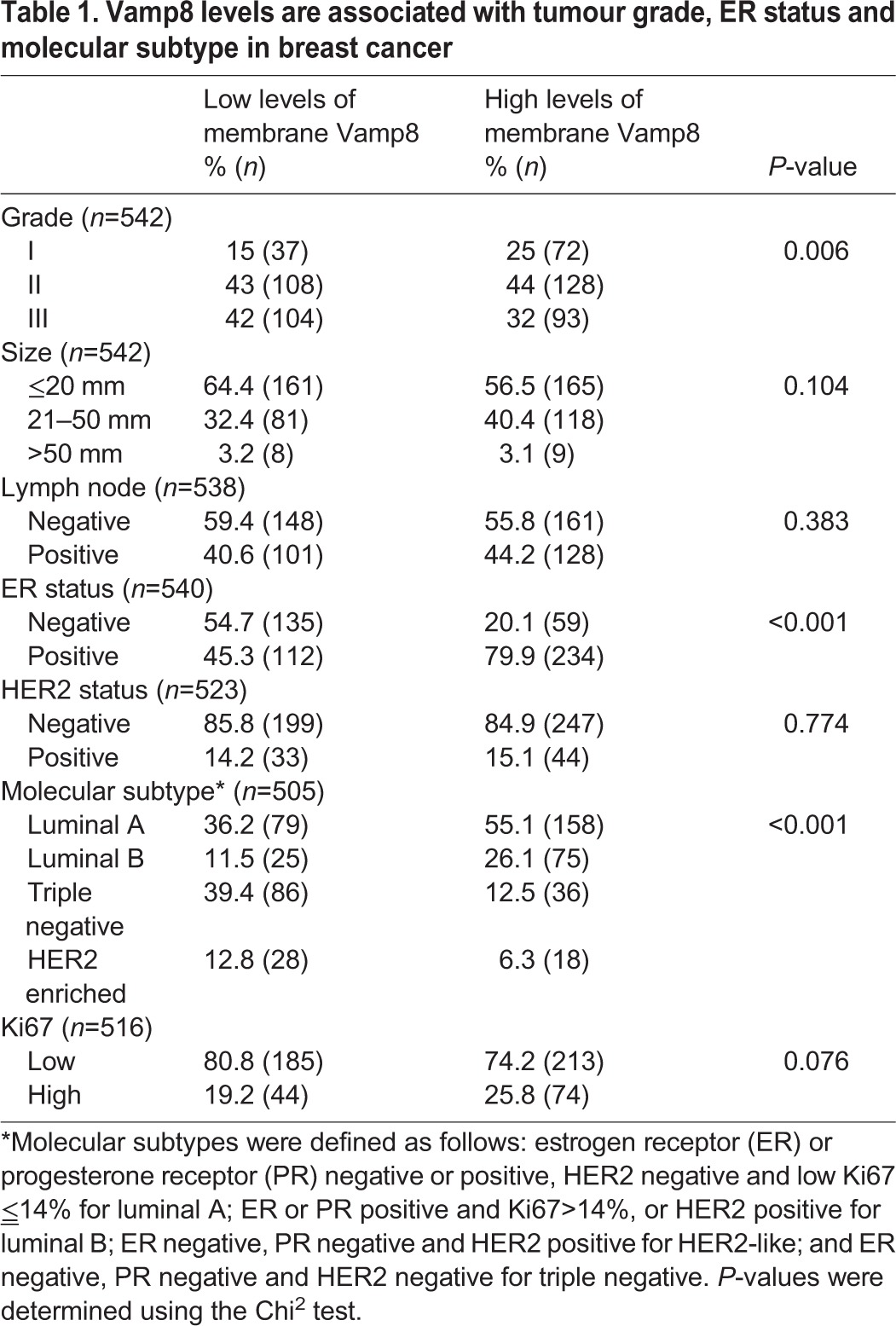
**Vamp8 levels are associated with tumour grade, ER status and molecular subtype in breast cancer**

### A SILAC-based MS screen reveals NRP2 to be a Rab17 cargo

**Fig. 5.**
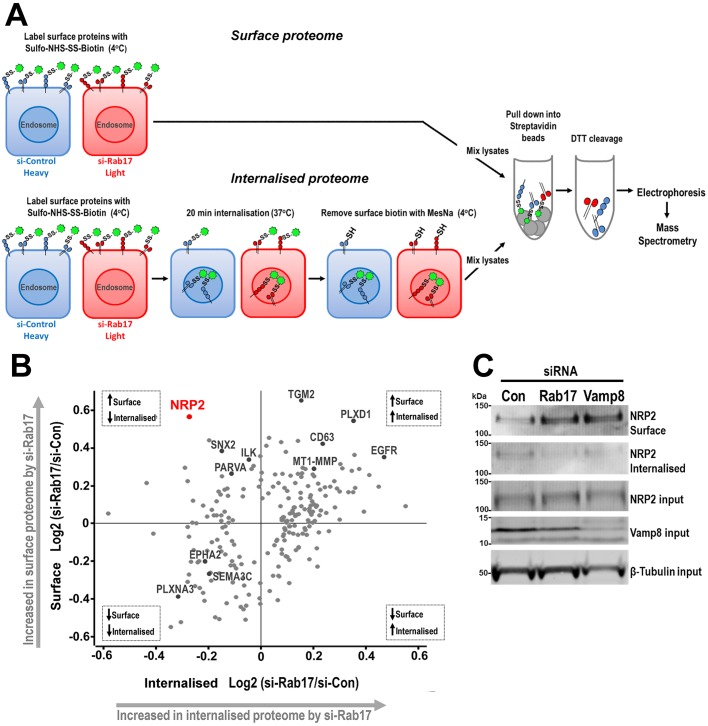
**SILAC-based MS screen reveals NRP2 to be a Rab17 cargo.** (A) Workflow of the SILAC-based MS approaches used for the comparative analysis of biotin-labelled surface (surface proteome) and internalised (internalised proteome) proteins in SILAC-labelled MDA-MB-231 cells. This experiment was performed in a forward (as indicated in this diagram) and reverse configuration in parallel. (B) Scatter plot showing the SILAC ratio between si-Rab17 and si-Control (logarithmic scale) cells for each significantly affected protein (grey dots). Upper-right and lower-left quadrants show proteins that are, respectively, up- and downregulated in both the cell surface and internalised proteome fractions. The upper-left and lower-right quadrants display proteins that are regulated in opposite directions at the cell surface and in internalised proteome fractions as indicated. *n*=2 SILAC experiments. Significance A statistical test, FDR of 5%, Perseus software. (C) MDA-MB-231 cells were transfected with siRNAs targeting Rab17 or Vamp8, or a non-targeting control (Con). Cell surface and internalised proteome fractions were prepared as indicated in A, and the presence of NRP2 in these was determined using western blotting. The cellular NRP2, Vamp8 and β-tubulin levels were also determined (input).

We hypothesised that Rab17 and Vamp8-regulated trafficking pathways might suppress invasion by either reducing delivery of pro-invasive receptors to the plasma membrane or by increasing recycling of anti-invasive cargoes. We designed a strategy (schematically summarised in Fig. 5A) that allowed us to identify alterations to receptor distribution between endosomes and the plasma membrane in an unbiased manner. To do this, we combined SILAC-based MS proteomics with compartment-specific protein biotinylation approaches. As before, we labelled control and Rab17-knockdown MDA-MB-231 cells with light-isotope and heavy-isotope SILAC amino acids, respectively (forward experiment), and with heavy-isotope and light-isotope amino acids, respectively (reverse experiment). We then surface-labelled these cells at 4°C with membrane-impermeant sulpho-NHS-SS-Biotin and isolated the fraction of plasma membrane proteins using streptavidin affinity chromatography – this complement of proteins we termed the ‘surface proteome’. In a separate set of culture dishes, we warmed SILAC-labelled surface-biotinylated cells to 37°C for 20 min to allow surface-labelled receptors to distribute between endosomes and the plasma membrane. As the sulpho-NHS-SS-Biotin reagent contains a disulphide linker that may be cleaved by reducing agents, we were able to remove biotin from receptors remaining at the cell surface through treatment with the membrane-impermeant reducing agent MesNa. The remaining endosomally localised biotinylated receptors were then isolated using streptavidin beads, and this fraction of proteins we termed the ‘internalised proteome’. The surface and internalised proteomes from the forward and reverse experiments were then analysed by performing high-resolution mass spectrometry. This provided unambiguous identification of 1640 surface proteome and 2272 internalised proteome components and, as expected, the majority of proteins isolated using this surface biotinylation approach were membrane and/or membrane-associated proteins (data not shown). We then looked for proteins whose relative abundance between surface and endosomal compartments was reproducibly (in the forward and reverse experiments) influenced by Rab17 knockdown. We categorised these according to whether their abundance was coordinately or differentially regulated at the cell surface and the endosome by Rab17 knockdown. The largest category constituted those proteins whose abundance was co-ordinately increased in the surface proteome and the internalised proteome by Rab17 knockdown (upper-right quadrant in Fig. 5B; Table S3). Prominent amongst these were the pro-invasive transmembrane matrix metalloproteinase MT1-MMP (also known as MMP14) and the receptor tyrosine kinase EGFR1, indicating that degradation of these receptors may be suppressed following Rab17 knockdown.

The purpose of this study was to identify new cargoes of Rab17 – i.e. proteins that were distributed differently between the cell surface and endosomes following siRNA knockdown of Rab17. We, therefore, focussed our attention on those proteins whose abundance moved in opposite directions in the surface proteome and internalised proteome following Rab17 knockdown (upper-left quadrant in Fig. 5B; Table S3). Interestingly, this analysis revealed neuropilin-2 (NRP2), which is a co-receptor for semaphorins and vascular endothelial growth factor (VEGF), to be the protein whose surface expression was most significantly increased (1.5-fold) at the cell surface and reduced (1.2-fold) in the internalised proteome when Rab17 was silenced (Fig. 5B). Western blotting confirmed that siRNA knockdown of Rab17 increased the quantity of NRP2 at the cell surface and decreased the endosomal pool of NRP2, whilst its total cellular content remained unchanged (Fig. 5C). Furthermore, we found that knockdown of Vamp8 evoked similar alterations to the plasma membrane and endosomal distribution of NRP2 to that elicited by knockdown of Rab17 (Fig. 5C).

### Trafficking of NRP2-positive vesicles is altered by suppression of Rab17 or Vamp8

**Fig. 6.**
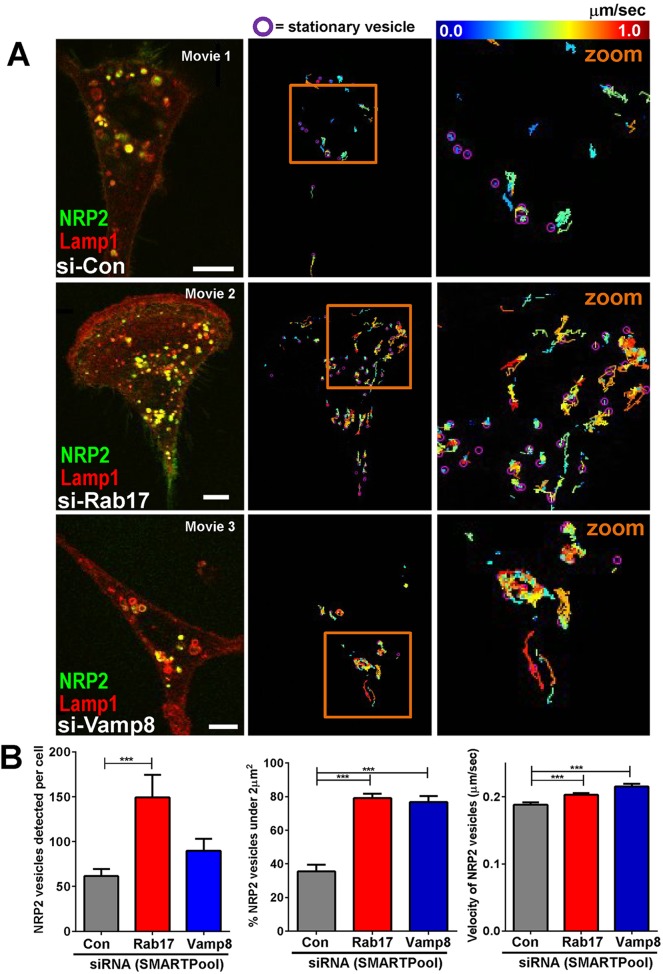
**NRP2 is relocated to a population of small motile transport vesicles following Rab17 or Vamp8 knockdown.** MDA-MB-231 cells were transfected with GFP-tagged NRP2 and mCherry-tagged Lamp1 in combination with siRNAs targeting Rab17 (si-Rab17), Vamp8 (si-Vamp8) or a non-targeting control (si-Con). Transfected cells were imaged by performing time-lapse fluorescence confocal microscopy. Movies were captured with 0.85 sec frame intervals over a period of 2.85 min. Stills were extracted from Movies 1–3, and GFP–NRP2 (green) and mCherry–Lamp1 (red) are displayed in the merged images (A; left panels). Scale bars: 10 µm. Movies were analysed using the TrackMate plug-in for ImageJ (Tinevez et al., 2016). The trackplots displayed in the centre panels (A) indicate the movement of GFP–NRP2 vesicles, with the speed of vesicle movement being denoted by a pseudocolour scale, and the positions of stationary vesicles are indicated using purple circles. The areas encompassed by the orange boxes in the centre panels are enlarged in the right-hand panels (A; zoom). The number, size and velocity of GFP–NRP2 vesicles were calculated, and these values are plotted in B. Values are mean±s.e.m., number of cells is 26 (si-Con), 23 (si-Rab17) and 20 (si-Vamp8), ***P<0.0001, Mann–Whitney test. Data are mean±s.e.m.

Our proteomic analysis indicated that NRP2 redistributes from endosomes to the cell surface following Rab17 knockdown, suggesting that the endosomal trafficking of this receptor is regulated by Rab17 and Vamp8. To visualise the trafficking of NRP2, we transfected MDA-MB-231 cells with GFP-tagged NRP2 in combination with the late endosomal marker mCherry–Lamp1 and performed fluorescence live-cell imaging followed by quantitative vesicle tracking. GFP–NRP2 was located at large Lamp1-positive late endosomes, which were located primarily in the perinuclear region, and quantitative analysis indicated that fewer than 40% of NRP2-positive structures occupied an area of less than 2 μm^2^ (Fig. 6A,B). Moreover, NRP2-positive vesicles had low motility, and many of the larger vesicles were largely stationary during the course of the movies. By contrast, following knockdown of either Rab17 or Vamp8, the number of smaller NRP2-positive vesicles increased dramatically (>70% of structures being less than 2 μm^2^), and quantitative tracking analysis indicated that the speed of their movement significantly increased (Fig. 6A,B). Moreover, the population of smaller vesicles that was evoked by knockdown of Rab17 or Vamp8 still overlapped substantially with Lamp1-containing structures. Taken together, these data indicate that Rab17 and Vamp8 act to restrict the movement of a late endosomal and/or lysosomal compartment in which NRP2 is concentrated and, following suppression of either of these two trafficking regulators, NRP2 is localised to a population of smaller and more motile late endosomes. This observation is consistent with increased mobilisation of NRP2-containing late endosomes from the perinuclear region to more peripheral cellular locations and to the plasma membrane.

### NRP2 is required for invasiveness that is evoked by suppression of Rab17 or Vamp8

**Fig. 7.**
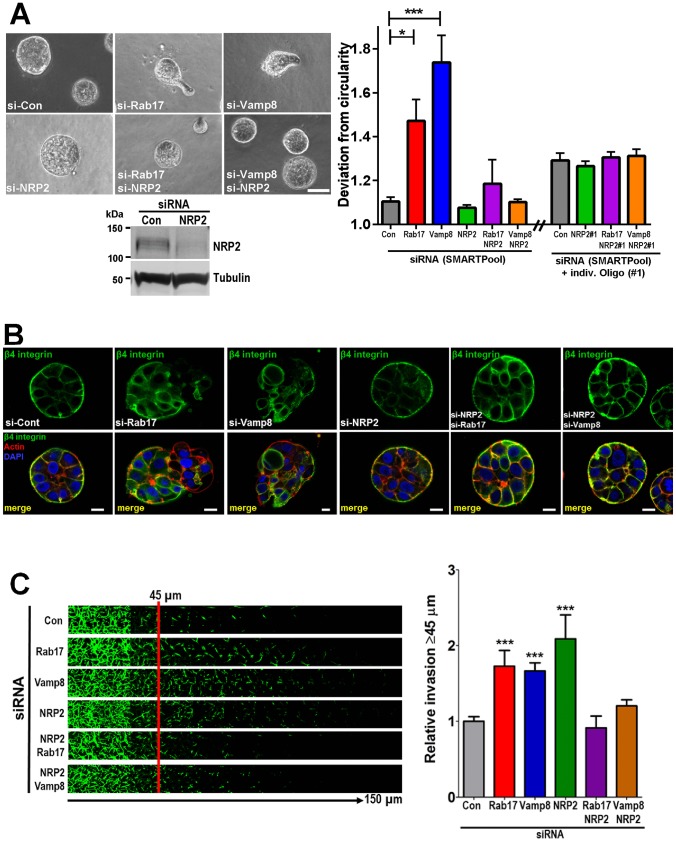
**NRP2 is required for invasiveness evoked by suppression of Rab17–Vamp8.** (A,B) MCF10ADCIS.com cells were transfected with siRNAs targeting Rab17 (si-Rab17), Vamp8 (si-Vamp8), NRP2 [si-NRP2 (SMARTPool) or an individual siRNA sequence (NRP2#1)] or a non-targeting control (si-Con) in the indicated combinations, and plated on a thin layer of Matrigel. After 3 days of culture, cells were visualised using phase-contrast microscopy (representative images shown) and the circularity of individual acini was determined. Scale bar: 50 μm. Circularity was measured in two independent experiments. Values in A are mean±s.e.m. (graph); *n*=21 (si-Con), *n*=15 (si-Rab17), *n*=12 (si-Vamp8), *n*=15 (si-NRP2), *n*=15 (si-Rab17 plus si-NRP2), *n*=12 (si-Vamp8 plus si-NRP2), *n*=71 (Con), *n*=143 (NRP2#1), *n*=140 (NRP2#1+Rab17) and *n*=121 (NRP2#1+Vamp8). Dunn's test for the multiple comparisons was performed (*P<0.05, ***P<0.001). (B) Cells analysed in A were then fixed, and laminin-V and β4 integrin were visualised by immunofluorescence [green signal and counterstained for F-actin (red signal) and nuclei (blue signal)]. Scale bars: 25 μm. The western blot in A indicates the efficiency of the NRP2 knockdown with tubulin used as a loading control. (C) MDA-MB-231 cells were transfected with siRNAs targeting Rab17 (si-Rab17), Vamp8 (si-Vamp8) and NRP2 (si-NRP2), or a non-targeting control (si-Con) in the indicated combinations, and invasiveness into fibronectin-supplemented Matrigel was determined using an inverted invasion assay as for Fig. 4A. Values are mean±s.e.m., *n*=6. Dunn's test for multiple comparisons was performed (***P<0.001). Data are mean±s.e.m.

Our MS analysis indicated that NRP2 redistributes from endosomes to the cell surface following Rab17 knockdown, suggesting that this receptor is a pro-invasive cargo whose recycling is opposed by Rab17 and Vamp8. We, therefore, provoked breast cancer cell invasiveness by knocking down Rab17 or Vamp8 and tested the requirement for NRP2 in this. Knockdown of NRP2 had no effect on the ability of MCF10DCIS.com cells to assemble basement-membrane-like structures and to form spherical acini when grown in 3D Matrigel cultures (Fig. 7A,B). However, siRNA knockdown of NRP2 (using either a SMARTPool siRNA or an individual siRNA sequence) opposed the ability of Rab17 and Vamp8 knockdown to drive basement membrane degradation and to disrupt acinar sphericity (Fig. 7A,B). Consistently, we found that knockdown of NRP2 completely opposed the ability of both Rab17 and Vamp8 knockdown to drive invasion of MDA-MB-231 cells into Matrigel plugs (Fig. 7C).

## DISCUSSION

The available database information concerning Rab17 and its interactors is insufficient to infer potential effectors and pathways through which this GTPase acts to suppress cancer invasiveness and progression. We, therefore, determined the Rab17 interactome and its influence on the cellular proteome in an unbiased manner. The use of high-accuracy MS-based approaches has enabled us to do this and to show that Rab17 has a close physical and functional relationship with the SNARE protein Vamp8. Rab GTPases are known to recruit specific effector proteins that bind to SNAREs, giving specificity to vesicle fusion (Angers and Merz, 2011). Nevertheless, to our knowledge, our study is the first to provide an indication that the stability and cellular levels of a SNARE protein may be controlled by a Rab GTPase. Moreover, because Vamp8 is the only component of the proteome that is significantly altered following Rab17 knockdown, the relationship between this GTPase and Vamp8 stability is likely to be highly selective. Our MS and immunofluorescence data indicate that Vamp8 is localised primarily to late endosomes and that its recruitment to this compartment is strongly dependent on Rab17. Thus, these data are consistent with a view that Rab17 acts primarily to recruit Vamp8 to late endosomes and, when Rab17 levels are low, Vamp8 becomes mislocalised and is subsequently degraded. We have found that neither proteosomal nor lysosomal mechanisms are responsible for degradation of mislocalised Vamp8 (data not shown). However, a report that caspases regulate Vamp8 expression and function in dendritic cells indicates the possibility that Rab17 could protect Vamp8 from caspase cleavage by localising it to late endosomes (Ho et al., 2009). Increased Vamp8 is primarily thought to promote homotypic fusion of either early or late endosomes (Antonin et al., 2000; Pryor et al., 2004). Endosomal compartments are constantly engaged in homotypic fusion and fission, and the relative rates at which these two processes occur dictate the size and identity of these compartments. Our observation that NRP2-containing late endosomes are large when Vamp8 levels are high is consistent with a role for this v-SNARE in homotypic fusion of these compartments. Thus, activation of ERK2, which leads to downregulation of Rab17 and, in turn, reduced Vamp8 levels might be expected to shift the homotypic fusion and fission balance of late endosomes in favour of fission to promote mobilisation of NRP2-containing late endosomes to the plasma membrane to drive invasion.

Rab17 expression has been known for a number of years to be associated with establishment of a polarised epithelial phenotype (Hunziker and Peters, 1998; Lütcke et al., 1993; Zacchi et al., 1998), and its downregulation is linked to loss of epithelial polarity (Lütcke et al., 1993). Our observations that downregulation of Rab17 accompanies events such as the DCIS to IDC transition and the disruption of acinar morphology (including breach of the basement membrane) are consistent with a role for Rab17 in maintaining epithelial morphology and opposing the epithelial to mesenchymal transition. Thus, it is probable that cargoes trafficked by Rab17 are involved in maintenance of epithelial polarity and, consistently, Rab17 has been shown to control trafficking events, such as transcytosis, that distinguish polarised epithelia (Hunziker and Peters, 1998; Zacchi et al., 1998). Vamp8 has been functionally linked to transcytosis (Pocard et al., 2007). Indeed, most evidence indicates that this SNARE, like Rab17, plays an important role in controlling trafficking events that support maintenance of epithelial polarity. In epithelial cells, Vamp8 is responsible for sorting apical cargoes, such as dipeptidyl peptidase IV (DPPIV), to the apical plasma membrane (Pocard et al., 2007). Many tumour cells retain some, but not all, of the characteristics of a polarised epithelium. For instance, comedo-forming tumour cells, such as MCF10DCIS.com, have a basement membrane, which corresponds to a basolateral region, but do not have a well-defined apical domain. Thus, it is interesting to speculate about the role played by the transcytotic machinery in partially polarised comedo-like organoids and other less-polarised cells. One possibility is that, in the absence of an apical domain (as encountered in MCF10DCIS.com cells), proteins such as Rab17 and Vamp8 might still function to move receptors that could potentially disturb basement membrane integrity away from the plasma membrane and into other compartments.

Characterisation of the endomembrane proteome is complicated by the fact that it is difficult, if not impossible, to obtain purified endosomal fractions that are uncontaminated by components from other cellular subcompartments. To overcome this, we have used a biotin-labelling approach, which allows enrichment of internalised protein cargo, and combined this with a quantitative SILAC method designed to highlight consistent changes in plasma membrane and endosomal distribution evoked in response to a defined pro-invasive stimulus. By focussing on these changes, interference from proteins that are non-specifically associated with plasma membrane and/or endosomal isolates is minimised. The receptor whose distribution was most significantly altered by Rab17 knockdown was NRP2. The neuropilins are well established as contributing to axonal guidance and angiogenesis (Raimondi and Ruhrberg, 2013); however, the roles played by these receptors in epithelial cells have received less attention. Neuropilins and the receptors with which they collaborate, such as VEGF receptors, are generally thought to be restricted to the apical and luminal domains of epithelial ducts to reduce their exposure to growth factor ligands that are abundant in the interstitium (Wild et al., 2012). Consistently, upregulation and/or mislocalisation of NRP1 and NRP2 to the basolateral region correlates with invasiveness and poor prognosis in a number of tumour types (Wild et al., 2012). Our data indicate that Rab17 and Vamp8 promotes removal of NRP2, and other potentially proinvasive receptors (such as MT1-MMP), from the basolateral membrane so that they can be routed to cellular locales where they will not disrupt basement membrane integrity. Thus, in normal epithelia, the role of a Rab17–Vamp8 complex might be to transcytose these receptors to the apical domain but, in partially transformed comedo-like tumours, Rab17 and Vamp8 strive to oppose the transition to invasive carcinoma by actively retaining NRP2 safely within the late endosomal system. Thus, Rab17 and Vamp8-dependent sequestration of NRP2 within the endosomes of tumour cells is likely to represent a remnant of the transcytotic system that still acts to suppress basement membrane disruption and the DCIS to IDC transition. Suppression of Rab17 levels through the activation of ERK2 signalling is likely to represent a key event in loss of the last vestiges of epithelial polarity, which allows tumours to invade and metastasise. Our use of novel MS proteomics to identify NRP2 as a cargo of the Rab17–Vamp8 pathway indicates that loss of control over trafficking of this receptor is a key event in the DCIS to IDC transition.

## MATERIALS AND METHODS

### Cell culture and transfection

MDA-MB-231 cells were cultured as described previously (von Thun et al., 2012). MCF10ADCIS.com cells were kindly provided by Prof. Philippe Chavrier (Institut Curie, Paris, France) and were cultured and transfected as described previously (Macpherson et al., 2014). siRNA oligonucleotides against Rab17 were SMARTpool, #1 5′-GAAGUGGCUCCGUGGGUAA-3′ or #2 5′-ACGCUGCGCUUCUGGUGUA-3′ (Dharmacon). siRNA oligonucleotides against Vamp8 and NRP2 were SMARTpool (Dharmacon). EGFP-C1-Rab17 was a gift from Jeremy Simpson (University College, Ireland). CD63–GFP and Rab11–GFP constructs were as described previously (Macpherson et al., 2014). GFP–NRP2 in pCMV6-AC-GFP was from Amsbio (MG223943), and GFP–Vamp8 was a gift from Thierry Galli (Institut Jaques Monod, Paris, France) [Addgene plasmid #42311 (Paumet et al., 2000)]. Cells were transfected using the Nucleofector system, as described previously (Macpherson et al., 2014). SILAC-labelled MDA-MB-231 cells were obtained by culturing in SILAC-DMEM lacking arginine and lysine (Life Technologies) supplemented with L-arginine and L-lysine (SILAC light) (Sigma-Aldrich) or [^13^C_6_][^15^N_4_] L-arginine and [^13^C_6_] [^15^N_2_] L-lysine (SILAC heavy; Cambridge Isotope Laboratories, Tewksbury, MA). 3D culture of MCF10DCIS.com cells and inverted invasion assays were performed as described previously (Macpherson et al., 2014).

### qPCR and western blotting

Primers against cDNA encoding GAPDH, Rab17 and Vamp8 were obtained from Qiagen (catalogue numbers QT01192646, QT00009590 and QT00086639, respectively). Primers against *NRP2* were ordered from Thermo Fisher Scientific (Wittmann et al., 2015), and quantitative PCR (qPCR) experiments were performed as described previously (von Thun et al., 2012). Antibodies recognising Vamp8 (catalogue number 104302, dilution 1:1000; Synaptic systems, Göttingen, Germany), Vamp7 (dilution 1:1000) and Vamp3 (dilution 1:1000) were a gift from Andrew Peden (University of Sheffield, UK). Antibodies against β-tubulin (Sigma-Aldrich, catalogue number T5201, dilution 1:1000), GFP (Abcam, Cambridge, UK, catalogue number AB6556, dilution 1:1000), catalogue number AB6556, dilution 1:1000), laminin-V (Millipore, catalogue number MAB19562, dilution (IF) 1:100). NRP2 (R&D, Abingdon, UK, catalogue number AF2215, dilution 1:1000) and ERK1 and ERK2 (ERK1/2; Santa Cruz, K-23, catalogue number B2607, dilution 1:1000) were used for western blotting.

### Recombinant protein expression, pulldown and site-directed mutagenesis

The Vamp8 sequence encoding amino acids 1–74 was sub-cloned into pGEX-6P-1 from GFP–Vamp8 plasmid using EcoR1 and Xho1 restriction sites. GST–Vamp8^cyto^ and GST–Rab17 were transformed into OneShot^®^ BL21(DE3) *E. coli* (Invitrogen), grown at 37°C for 6 h and then induced with isopropyl β-D-1-thiogalactopyranoside (IPTG) for 16 h. Bacteria were lysed by sonication in PBS and proteins purified using glutathione–Sepharose beads (GE Healthcare). Vamp8 was subsequently cut from the beads with PreScission protease (GE Healthcare) with 1.6 units per 10 µl of cleavage buffer (50 mM Tris-HCl, pH 6.8, 150 mM NaCl, 1 mM EDTA, 0.01% Triton X-100, 1 mM DTT). The resulting protein was quantified using NuPAGE Tris-acetate gel, and equal quantities of Vamp8 and Rab17 were incubated for 16 h at 4°C. The beads were then washed twice with PBS, and the input and beads run on NuPAGE Tris-acetate gel, and whole protein levels were analysed with InstantBlue™ Coomassie stain (Expedeon).

Site-directed mutagenesis was performed using QuikChange Lightning kit (Agilent) to create GST–Rab17Q77L (forward 5′-TGTGGTACTTCTTCCAGGCCAGCTGTGTCC-3′ and reverse 5′-GGACACAGCTGGCCTGGAGAAGTACCACA-3′), and the two rescue vectors resistant to Rab17 si-#1, GFP–Rab17 T81C_C84G_C87A_G90T (forward 5′-GCCAAGCTGGACTTACCAACTGACCCGCTTCCCAGGAGAACCAG-3′, reverse 5′-CTGGTTCTCCTGGGAAGCGGGTCAGTTGGTAAGTCCAGCTTGGC-3′) and GFP–Vamp8 resistant to Vamp8 si-#2 C234T_T237G_C240A_C243T (forward 5′-CTGGTGGAAGAACGTGAAGATGATTGTTCTGATATGTGTGATTGTTTTTATCATCATCCTCTTC-3′, reverse 5′-GAAGAGGATGATGATAAAAACAATCACACATATCAGAACAATCATCTTCACGTTCTTCCACCAG-3′).

### Surface and internalised proteome analysis

SILAC-labelled MDA-MB-231 cells were transfected with siRNAs targeting Rab17 or a non-targeting control and plated into 10 cm plastic dishes. 48 h later, cells were surface-biotinylated through incubation with 0.2 mM sulpho-NHS-SS-Biotin (Thermo Fisher Scientific) in PBS at 4°C for 1 h. To study the internalised proteome, surface-biotinylated cells were transferred to SILAC-DMEM (Gibco, Life Technologies) at 37°C for 20 min to allow internalisation of labelled protein, and then biotin remaining at the cell surface was removed by incubation with a cell-impermeant reducing agent (MesNa) 20 mM (Fluka, Sigma-Aldrich) in Tris-buffered saline (pH8.6) for 50 min at 4°C. Remaining MesNa was quenched by incubation with iodoacetamide (IAA; 20 mM Sigma-Aldrich). Cells were lysed in 200 mM NaCl, 75 mM Tris, 15 mM NaF, 1.5 mM Na3VO4, 7.5 mM EGTA, 1.5% Triton X-100, 0.75% Igepal CA-630, 0.5 mg/ml leupeptin (Melford, Suffolk, UK), 0.25 mg/ml aprotinin (Sigma-Aldrich) and 5 mM 4-(2-aminoethyl)benzynesulphonyl fluoride (AEBSF; Melford), and lysates were cleared by centrifugation at 10,000 ***g*** for 10 min at 4°C. The supernatants from the heavy-isotope and light-isotope SILAC-labelled cells were then mixed, and biotinylated proteins were captured by incubation with streptavidin–agarose beads (Upstate, Millipore) for 1 h at 4°C with constant rotation. Following extensive washing, proteins that were specifically associated with the beads were eluted through reduction with 0.1 M DTT (0.1 M) in a Tris buffer (pH 7.5).

### MS analysis

Proteins were separated on 4–12% gradient NuPAGE Novex Bis-Tris gel (Life Technologies), visualised by staining of the gels with Coomassie Blue and digested in-gel (8 gel slices were cut for the surface proteome and internalised proteomes; 5 gel slices for Rab17 immunoprecipitation) using trypsin (Shevchenko et al., 2007) and desalted as described previously (Rappsilber et al., 2007). For the proteome analysis of cells that had been silenced for Rab17, proteins were trypsin-digested using the filter-aid sample preparation (FASP) method, and 50 µg of peptides were separated by using strong anion exchange chromatography on StageTip as previously described (Wisniewski et al., 2009). Digested peptides were loaded onto an EASY-nLC instrument connected online to an LTQ-Orbitrap Elite mass spectrometer (Thermo Fisher Scientific). Peptides were separated using a 20 cm fused silica emitter (New Objective) packed in-house with reversed-phase Reprosil Pur Basic 1.9 µm (Dr Maisch GmbH) and eluted with a flow of 200 nl/min from 5% to 20–30% of buffer containing 80% acetonitrile in 0.5% acetic acid over a 90 min (190 min gradient for Rab17-knockdown cells) linear gradient. The full scan MS spectra were acquired at a resolution of 120,000 at *m*/*z* 400. The top ten most intense ions were sequentially isolated for fragmentation using high-energy collision dissociation and recorded at a resolution of 15,000. Data were acquired with Xcalibur software (Thermo Fisher Scientific).

### MS data analysis

The MS files were processed with the MaxQuant software (Cox and Mann, 2008) version 1.3.7.4 (proteome) and 1.3.8.2 (internalised proteome and interactome), and searched with the Andromeda search engine (Cox et al., 2011) against the human UniProt database (release-2012 01, 88,847 entries). The common reverse and contaminant hits (as defined in MaxQuant output) were removed. Only protein groups identified with at least one uniquely assigned peptide were used for the analysis. For the SILAC experiments, the SILAC ratios between light-isotope and heavy-isotope peptides were calculated using MaxQuant. Protein groups were considered reproducibly quantified if identified and quantified in the forward and reverse experiments. For label-free quantification, proteins were quantified according to the label-free quantification algorithm available in MaxQuant (Cox et al., 2014).

#### SILAC analysis of the surface proteome and internalised proteome

From the filtered list of quantified proteins, hits with opposite regulation (opposing signs when the SILAC ratio was expressed as log_2_) in the two experiments were excluded. To identify differentially trafficked proteins upon Rab17 knockdown, proteins belonging to the 1st or 3rd quartiles of the SILAC ratio distribution of the forward and reverse experiments were considered. This statistical approach allowed us to select proteins with small but reproducible (in both forward and reverse SILAC experiments) changes in localization.

#### SILAC analysis of the Rab17-knockdown proteome

For forward and reverse experiments, protein groups with significant SILAC ratios were determined according to the significance B test (Cox et al., 2011), using 5% as the false detection rate (FDR). The significance B test identifies outliers based on the standard deviation of the protein SILAC ratio of the main distribution and the protein abundance (intensity measured at the mass spectrometer). The significance B test was chosen because, as typically shown for SILAC datasets (Cox et al., 2011), the distribution of the logarithmic SILAC ratios was dependent on the protein intensity, where the spread of nonregulated proteins was higher for low- compared to high-abundance proteins. For this reason, for low-abundance proteins to be significant, a higher deviation from the SILAC ratio main distribution is required. Conversely, for high-abundance proteins, a smaller deviation is required.

#### Label-free analysis of the interactome

Three independent replicates were generated per condition (control and Rab17 immunoprecipitation), and significantly enriched proteins were selected using a Welsh-test-based analysis (5% FDR). The Welsh test is particularly suitable for this kind of analysis, where control and Rab17-immunoprecipitated samples could recover different numbers and amounts of interacting protein, because this test is designed for studies where the two samples have unequal variances and unequal sample sizes.

### Immunofluorescence and immunohistochemistry

Antibodies against CD63 (PeliCluster, M1544, dilution 1:200) and Lamp2 (BD Pharmingen, 555803, dilution 1:100), dilution 1:200 phalloidin–Alexa-Fluor-546 (Thermo Fisher Scientific, dilution 1:400), laminin V (Millipore, catalogue number MAB 19562, dilution 1:100), and β4 integrin (BD Biosciences, catalogue number 555722, dilution 1:100) were used for immunofluorescence. Cells were stained and imaged using either 64× or 60× objectives with an inverted confocal microscope (Fluoview FV1000; Olympus). To quantify localisation, the confocal images were analysed using colocalisation2 plugin in ImageJ, and the Pearson's coefficient was plotted, which indicates positively correlated pixels. Immunohistochemical staining was performed as described previously (Dozynkiewicz et al., 2012; Macpherson et al., 2014) – the antibody recognising Vamp8 was from Synaptic systems (Göttingen, Germany, catalogue number 104302, dilution 1:100).

### Live-cell imaging

Cells were imaged using a 60× objective and an inverted confocal microscope (Fluoview FV1000; Olympus). Movies were captured with 0.85 s frame intervals over a period of 2.85 min. Tracking of GFP–NRP2 vesicles was performed using TrackMate plugin (Tinevez et al., 2016) and size data were collected manually, both using ImageJ software.
